# Paramedic students’ confidence and satisfaction with clinical simulations of an emergency medical care programme in South Africa: A cross-sectional study

**DOI:** 10.4102/hsag.v26i0.1522

**Published:** 2021-04-28

**Authors:** Peter T. Sandy, John T. Meyer, Oluwaseun S. Oduniyi, Azwihangwisi H. Mavhandu-Mudzusi

**Affiliations:** 1 Department of Nursing and Allied Health, Buckinghamshire New University, London, United Kingdom; 2 Department of Emergency Medical Sciences, Faculty of Health and Wellness, Cape Peninsula University of Technology, Cape Town, South Africa; 3 Department of Agriculture and Animal Health, Faculty of Agriculture and Environmental Science, University of South Africa, Florida Campus, Roodepoort, South Africa; 4 Department of Health Studies, Faculty of Human Sciences, University of South Africa, Pretoria, South Africa

**Keywords:** clinical simulation, emergency medical care, paramedic, satisfaction, self-confidence

## Abstract

**Background:**

There has been an increase in the use of clinical simulations as instructional tools in healthcare education. This is because of their role in ensuring patients’ safety and quality-care provision.

**Aim:**

This study investigated the paramedic students’ satisfaction and self-confidence in the clinical simulation of an emergency medical care programme.

**Setting:**

The study was conducted at the Durban University of Technology in the KwaZulu-Natal Province of South Africa. The paramedic students’ satisfaction and self-confidence in the clinical simulation of an emergency medical care programme were the focus of the study.

**Methods:**

The study used a cross-sectional research design. A convenience sampling method was used to select the 83-paramedic students who participated in the study. Data were collected between July and September 2017 using a structured questionnaire. Descriptive statistics (frequencies and percentages and Spearman’s rank-order correlation coefficient) and an inferential test, ordinal logistic regression analysis, were used for data analysis.

**Results:**

High levels of paramedic students’ satisfaction and self-confidence in simulation activities were reported. Generally, the paramedic students’ demographics were associated with the satisfaction and self-confidence variables with *p*-values ≤ 0.04. Emergency medical care training undertaken by the paramedic students was significantly associated with self-confidence (*p* = 0.00).

**Conclusion:**

Clinical simulation can bridge the theory-practice gap for paramedic students. It is a hands-on approach that promotes students learning of clinical skills through reflection.

## Introduction and background

Clinical simulation is an artificial representation of real clinical scenarios to achieve educational goals through learning-by-doing and reflection (Özkalp & Saygılı 2015). Simply, it is an educational activity that uses simulation aides to replicate as close as possible real clinical scenarios (Sarman & Pardi 2019). These explanations indicate that clinical simulation is a ‘student-centred and hands-on’ approach that considers the learning needs and styles of learners.

Over the past two decades, healthcare education witnessed an increase in the use of clinical simulations in its instructional designs (Karkada et al. 2019). This is a function of the role clinical simulation plays in ensuring quality care provision and patients’ safety, given that it enables learners to repetitively practise clinical skills to specific levels of proficiency in risk-free environments (Rodriguez et al. 2017). The notion of risk-free environments suggests that students can make mistakes and learn from them without the fear of harming patients. Hence, following any clinical simulation experience, students are often encouraged by facilitators to clarify any concerns they might have. Such a collaborative approach to learning enables students to consolidate the insights gained from the clinical simulation experience, and link theory with practice. The rationale here is that skill acquisition is often highest when teaching and learning take place in settings comparable to real-life situations (Bransford, Brown & Cocking 1999). As a result, clinical simulations are commonly used in healthcare education to develop students’ cognitive, psychomotor and affective skills (Alfes 2011). For example, in the United States of America, the National Council of State Board for Nursing endorsed the use of clinical simulation in the training of new nurses to develop their clinical competence (Rodriguez et al. 2017). Similarly, countries in Europe (e.g. the United Kingdom and Germany) and Asia (e.g. India and Malaysia) endorsed clinical simulation for enhancing clinical and decision-making skills of healthcare workers, including paramedics (Burbach et al. 2019). Despite this, simulation education is not free from challenges. In addition to mannequins not being able to provide verbal and non-verbal feedback, healthcare students have reported feelings of anxiety evoked by scenarios used during simulation activities (Weller 2004). Being exposed to feelings of anxiety, especially over prolonged periods, negatively impacts on learning. Hence, healthcare students have reported dissatisfaction with clinical simulation (Sarman & Pardi 2019).

Nevertheless, clinical simulation is widely valued and forms an integral component of paramedic student training in several regions of the world, including Europe, Africa, Asia and the United States of America (Karkada et al. 2019). This is because it creates a satisfying, safe and engaging space where active and meaningful learning of critical thinking and clinical skills take place. Acknowledging this, it is not surprising for this instructional design to be associated with paramedic students’ enhanced satisfaction, competence, self-confidence, and ability to safely apply learnt skills and knowledge in clinical practice (Rodriguez et al. 2017). The outcome of a study by Williams et al. (2016) that compares learners’ satisfaction with simulation between Australian and Jordanian paramedic university students confirms this. Even though there were differences in satisfaction levels, the paramedic students were in the main satisfied with clinical simulation regardless of their socio-cultural backgrounds. Despite this, most research on clinical simulation focus on its effectiveness in achieving training goals with areas such as learners’ confidence, competence and satisfaction with stimulation training much neglected (Adamson, Kardong-Edgren & Willhaus 2013). Hence, this study investigated the paramedic students’ self-confidence and satisfaction with clinical simulations of an emergency medical care (EMC) programme at a university in South Africa.

### Theoretical framework

The cultural-historical activity theory (Engeström 1987) commonly known as CHAT, serves as the basis for this study. It posits that a relationship exists between the human mind (what people think and feel) and activity (what people do). Basically, people’s behaviours are influenced by their thoughts and emotions. Given this, CHAT claims that people act collectively, learn by doing, and communicate in and through their actions. It adds that people make, employ and adopt tools of all kinds to learn and communicate. Simulators are some examples of tools that enable paramedic to interact, practice and learn skills, and develop knowledge for the provision of safe and effective care. From a CHAT perspective, knowledge is never isolated from its historical context but is socially and discursively constructed (Engeström 1987). This indicates that knowledge and context are intertwined and inseparable. Cultural-historical activity theory, therefore, stresses that humans are enculturated, and their cultural values and resources shape their behaviours. Thus, people’s behaviours must always be viewed in the light of historical trajectories in which their behaviours take place, given that cultures are grounded in histories and evolve over time. In light of this, CHAT claims that community is central to the process of making and interpreting the meaning of learning and behaviours of people. The community here is the paramedic students of the emergency medical programme.

## Materials and methods

### Study area

The study was conducted at the Durban University of Technology in the KwaZulu-Natal Province of South Africa. It is one of the four universities of technologies in South Africa that offer a 4-year bachelor’s degree programme in EMC for paramedics.

### Design

This is a quantitative study that utilised a cross-sectional research design. This design was chosen for several reasons. It is appropriate for analysing and interpreting data characteristics collected from a sample of a population within a specific period with no follow-up required (Polit & Beck 2014). It assists researchers in describing sample characteristics, which in this case, relate to paramedic students’ self-confidence and satisfaction levels with the clinical simulation of the EMC programme at the Durban University of Technology.

### Population, eligibility criteria, and sample

The study population was all undergraduate paramedic students (103), enrolled on the bachelor’s degree of the EMC programme at the Durban University of Technology during the time of data collection. Eligibility for the recruitment of participants was based on these criteria:

- Paramedic students on the EMC programme with a minimum of 1-year experience of using simulators at the Durban University of Technology.
- Paramedic students in the second and third year of the EMC programme at the Durban University of Technology.

All the paramedic students had experience of using a simulator, a type of manikin, which was the Laerdal ® patient simulator of high to low fidelity. However, 20 of the 103 paramedic students were in the first year of the EMC programme and were therefore, excluded from participating in the study. This means only 83 of the paramedic students were eligible for participation.

A convenience sampling method was adopted to recruit the participants. One of the investigators (J.M.) met with potential participants during the simulation lessons following receipt of ethical clearance from the Durban University of Technology Ethics Committee to conduct the study. In addition to providing an information leaflet to each potential participant, the study was explained to them, including its aim and benefits. They were also informed that participation was voluntary and anonymous, and they have the freedom not to participate should they so choose. A request was then made, for them to express their willingness to participate by signing a consent form. The eligible potential participants (83) each signed a consent form, and thus constituted the study sample.

### Data collection

Data were collected using the Satisfaction and Self-confidence Questionnaire (SSCQ) developed by the researchers. The SSCQ is divided into two sections, A and B. Section A consists of 11 items designed to elicit participants’ demographic data, such as age and gender. Section B consists of two, five-point Likert scales, satisfaction and self-confidence with five items and eight items, respectively. The SSCQ was piloted and the outcome resulted in its revision. The revised questionnaire was then sent to two experts in simulation-based education for comments. No comments were provided, as they were satisfied with the questionnaire. The questionnaire was then subjected to an internal consistency test for the two scales. The Cronbach’s alpha for the satisfaction items was 0.92 and 0.83 for the self-confidence items.

Data were collected between July 2017 and September 2017 after participants were recruited. The questionnaire was administered to each participant in the simulation laboratory after every simulation session at the Durban University of Technology. Each simulation session consisted on average of four participants. Data were collected from 21 simulation sessions within the data collection period, and the small number of participants per simulation session allowed for privacy and for the participants to sit 4 metres apart when completing the questionnaire. John Meyer (JM) was available to assist in completing the questionnaire when indicated. The completion of the questionnaire took on an average of 40 min. Participants handed all completed questionnaires in person to JM, and each of the questionnaires was assigned a unique number to assure anonymity and confidentiality.

### Data analysis

Data were analysed by JM and O.O. The Statistical Package for the Social Sciences (SPSS) version 25 was employed for data analysis. Initially, the demographic and Likert scales’ data were entered into the SPSS spreadsheet, and then analysed using descriptive and inferential statistics. Concerning descriptive statistics, frequencies and percentages were calculated on the demographic data and participants’ responses to the satisfaction and self-confidence Likert scales. The items (statements) of the Likert scales were scored five for ‘strongly agree’ down to one for ‘strongly disagree’. The ‘strongly agree’ and ‘agree’ items were combined to imply ‘agreement’ and negative statements ‘strongly disagree’ and ‘disagree’ items were combined and interpreted as ‘disagreement’. Spearman’s rank-order correlation coefficient (*r*) was used to examine associations between the satisfaction and self-confidence variables, and participants’ demographic characteristics. An ‘*r*’ value of 0.1–0.3 is considered a weak relationship, 0.3–0.5, a moderate relationship and over 0.5, a strong relationship (Grove, Burns & Gray 2013).

The inferential statistical test used was the ordinal logistic regression analysis. This was performed to determine factors independently associated with students’ satisfaction and self-confidence levels following simulation activities. The results of this analysis were expressed as odds ratios (OR) and 91% confidence intervals (CI).

### Ethical considerations

Ethical clearance was obtained from the University of South Africa (Ref: 2017/03/15/49937154/19/MC).

## Results

### General characteristics of the paramedic students

The demographic characteristics of the paramedic students are summarised in Table 1. A total of 83 paramedic students completed the questionnaires, 47 (56.6%) were in the 18–23 age range, 56 (67.5%) were men and 27 (32.5%) were women. Seventy-nine (95%) of the paramedic students disclosed their race, most of them 41 (49.4%) were African people, 22 (26.5%) were white people, 14 (16.9%) were Indian people and 1 (1.2%) were mixed race people, the majority of the paramedic students 74 (89.2%) were not married, however, 9 (10.8%) were married.

**TABLE 1:** General characteristics of students (***N*** = 83).

Demographic information	Frequency	
	*n*	%
**Age (years): (*n* = 83)**		
18–23	47	56.6
24–29	18	21.7
30–35	12	14.5
36–41	3	3.6
42–53	3	3.6
**Gender: (*n* = 83)**		
Male	56	67.5
Female	27	32.5
**Race (*n* = 83)**		
African people	41	49.4
Mixed race people	1	1.2
Indian people	14	16.9
White people	22	26.5
Prefer not to disclose	5	6.0
**Marital status (*n* = 83)**		
Married	9	10.8
Single	74	89.2
**Admission to the EMC Programme based on RPL (*n* = 83)**		
Yes	21	25.3
No	62	74.7
**Simulation experience (*n* = 83)**		
Yes	83	100.0
No	0	0.0
**Years of simulation experience (*n* = 46)**		
Less than 1 year to 1 year	28	60.9
2–4 years	16	34.8
Over 4 years	2	4.3
**Emergency medical service (EMS) training before joining the EMC programme (*n*=83)**		
None	38	45.8
First Aid/responder	10	12.0
Emergency care technician (ECT)	5	6.0
Ambulance emergency assistant (AEA)	11	13.3
Basic ambulance assistant (BAA)	12	14.5
Advanced life support practitioner (ALS)	2	2.4
Fire fighter 1 or 2 training and others (BAA, AEA, ECT, ALS, First Aid)	5	6.0
**Obtained EMS related training other than that offered by the EMC programme (e.g. BLS, PALS, ACLS etc.) (*n*=83)**		
Yes	29	34.9
No	54	65.9
**EMS related training undertaken (*n* = 29)**		
Basic life support (BLS)	16	55.1
Paediatric advanced life support (PALS)	0	0.0
Advanced cardiac life support (ACLS)	1	3.4
International trauma life support (ITLS)	1	3.4
Disaster management course	0	0.0
Emergency dispatchers’ course	1	3.4
More than one type of EMC related training (BLS, PALS, ACLS, ITLS, EMD)	10	34.5
**Occupation (work and study) (*n* = 83)**		
Yes	25	30.1
No	59	69.9
**Work description (*n* = 25)**		
EMS related	22	-
Non-EMS related	3	12.0

EMC, emergency medical service.

Concerning admission, most of the paramedic students 62 (74.7%) had the basic requirements for enrolment to the EMC programme, whilst 21 (25.3%) gained access to the programme through the recognition of prior learning route. In relation to simulation experience, all the paramedic students 83 (100%) had an experience in simulation, 2 (4.3%) had over 4 years of experience, 16 (34.8%) had 2–4 years of experience and 28 (60.9%) had a year experience.

With regard to the type of emergency medical service training received before the EMC programme, most of the paramedic students 38 (45.6%) had no prior emergency medical service training, 12 (14.5%) had the basic ambulance assistant training, 11 (13.3%) had the ambulance emergency assistant training, 10 (12%) had the first aid/responder training, 5 (6%) had the emergency care technician and fire fighter 1 or 2 training, and 2 (2.4%) had the advanced life support practitioner training.

Fifty-four (54%) of the paramedic students only had the emergency medical service training of the EMC programme. Thus, 29 (34.9%) of the students had another emergency medical service training in addition to that of the EMC programme. The majority of these students 16 (55.1%) had basic life support training, followed by 10 (34.5%) who had more than one type of EMC-related training. One (3.4%) had training in either the advanced cardiac life support or international trauma life support or the emergency dispatchers’ course.

As regards employment, the majority of the paramedic students 59 (69.9%) did not work and study, and 25 (30.1%) were in employment whilst studying. Most of the latter category of students 22(88%) was employed in the emergency medical services and 3 (12%) worked in the non-emergency medical services.

## Satisfaction and self-confidence in simulation learning

### Satisfaction with simulation learning

Table 2 shows the paramedic students’ levels of satisfaction with the simulation activities. It reveals that 52 (62.7%) of the paramedic students agreed that the teaching methods used in the simulation were helpful and effective, whilst 14 (16.9%) were opposed to that view. Fifty-five (66.3%) agreed that the simulation was well resourced with activities for learning clinical skills, but 17 (20.5%) were at odds with this opinion (question 2.2). Fifty-five (66.3%) noted that the teaching materials used during simulation motivated and helped them to learn, however, 14 (16.9%) disagreed (question 2.4). Forty-three (51.8%) enjoyed how their lecturers delivered the simulation training, whilst 40 (48.2%) noted that the delivery of the simulation by lecturers was consistent with their learning styles.

**TABLE 2:** Responses to the Likert scales.

Question		Number of response	Agree		Undecided		Disagree	
		*N*	Freq.	%	Freq.	%	Freq.	%
**Satisfaction with simulation learning**								
Q2.1	The teaching methods used in this simulation were helpful and effective.	83	52	62.7	17	20.5	14	16.9
Q2.2	The simulation provided me with a variety of learning materials and activities to promote my learning of clinical practical skills.	83	55	66.3	11	13.3	17	20.5
Q2.3	I enjoyed how my lecturer delivered the simulation training.	83	43	51.8	21	25.3	19	22.9
Q2.4	The teaching materials used in this simulation were motivating and helped me to learn.	83	55	66.3	14	16.9	14	16.9
Q2.5	The way my lecturer(s) taught the simulation was suitable to the way I learn.	83	40	48.2	20	24.1	23	27.7
**Self-confidence in simulation learning**								
Q2.6	I am confident that I am mastering the content of the simulation activity that my lecturers presented to me.	83	35	42.2	21	25.3	27	32.5
Q2.7	I am confident that this simulation covered critical content necessary for the mastery of clinical practical module.	83	40	48.2	28	33.7	15	18.1
Q2.8	I am confident that I am developing the skills and obtaining the required knowledge from this simulation to perform necessary tasks in a clinical setting.	83	47	56.6	26	31.3	10	12.0
Q2.9	My lecturers used helpful resources to teach the simulation.	83	50	60.2	18	21.7	15	18.1
Q2.10	It is my responsibility as a student to learn what I need to know from this simulation activity.	82	56	68.3	10	12.2	16	19.5
Q2.11	I know how to get help when I do not understand the concepts covered in the simulation.	83	61	73.5	16	19.3	6	7.2
Q2.12	I know how to use simulation activities to learn critical aspects of clinical skills.	82	55	67.1	17	20.7	10	12.2
Q2.13	It is the lecturer’s responsibility to tell me what I need to learn of the simulation activity content during class time.	82	56	68.3	13	15.9	13	15.9

### Self-confidence in simulation learning

Table 2 shows the paramedic students’ levels of confidence with the simulation activities. It shows that 35 (42.2%) of the students agreed that they had mastered the content of the simulation activity that their lecturers presented to them, and 27 (32.5) disagreed with the same. Added to this, 40 (48.2%) of the students agreed that the simulation lessons covered the critical content necessary for the mastery of the clinical practice module, 28 (33.7%) were undecided and 15 (18.1%) disagreed. As regards clinical skills, 47 (56.6%) of the paramedic students were in agreement that the simulation lessons enabled them to acquire the skills and knowledge necessary for clinical practice. However, 10 (12%) of the students disagreed and 26 (31.3%) were undecided.

With regard to teaching resources, 50 (60.2%) of the paramedic students agreed that the teaching resources for the simulation training were helpful, 15 (18.1%) disagreed and 18 (21.7%) were undecided. Most of the paramedic students 56 (68.3%) agreed that it was their responsibility to learn what they needed to know from the simulation activity, but 16 (19.5%) disagreed. Whilst this was the case, 56 (68.3%) of the students also agreed that it was the lecturer’s responsibility to tell them what they needed to learn from the simulation lessons, 13 (15.9%) disagreed and 13 (15.9%) were undecided. However, 61 (73.5%) of the paramedic students agreed that they knew how to get help when they did not understand the concepts covered in the simulation lessons, and only 6 (7.2%) disagreed. The majority of paramedic students 55 (67.1%) agreed that they knew how to use simulators to learn critical clinical skills, but 10 (12.2%) disagreed.

### Relationships between the paramedic students’ demographic characteristics, and satisfaction and self-confidence variables

#### Demographic characteristics and satisfaction variables

Table 3 shows the associations between the paramedic, the students’ demographic characteristics and satisfaction variables. A positive and significant but weak association was found between age and the satisfaction variable, ‘I enjoyed how my lecturer delivered the simulation training’ (*r* = 0.233, *p* = 0.04). The satisfaction variable, ‘The way my lecturer(s) taught the simulation was suitable to the way I learn’, also showed a positive, significant and weak association with age (*r* = 0.396, *p* = 0.00). A significant association was also found between years of simulation experience and the following satisfaction variables, ‘The teaching methods used in this simulation were helpful and effective’ (*r* = –0.336, *p* = 0.03), and ‘I enjoyed how my lecturer delivered the simulation training’ (*r* = –0.346, *p* = 0.03). The association between the medical service-related training of the EMC programme and the satisfaction variable, ‘The teaching methods used in this simulation were helpful and effective’ was reported to be statistically significant (*r* = –0.343, *p* = 0.02). The satisfaction variable, ‘The way my lecturer(s) taught the simulation was suitable to the way I learn’, showed a statistically significant and weak association with the work and study variable (*r* = –0. 227, *p* = 0.04).

**TABLE 3:** Spearman’s rank correlation coefficients (*r*) for demographic characteristics and satisfaction and self-confidence variables (*N* = 83).

Variable	Age		Gender		Race		Marital status		Admission to the EMC programme and RPL		Simulation experience		Years of simulation experience		EMS related training of the EMC Programme		EMS related training not offered by the EMC Programme		EMS related training undertaken		Work and study		Work description	
	*r*	*p*	*r*	*p*	*r*	*p*	*r*	*p*	*r*	*p*	*r*	*p*	*r*	*p*	*r*	*p*	*r*	*p*	*r*	*p*	*r*	*p*	*r*	*p*
**Satisfaction**																								
Q2.1	0.193	0.08	−0.024	0.84	−0.103	0.36	−0.086	0.44	0.084	0.46	0.025	0.83	-.336	0.03	−0.343	0.02	−0.071	0.53	−0.019	0.87	0.073	0.52	0.009	0.95
Q2.2	0.057	0.62	0.004	0.97	−0.108	0.34	−0.084	0.46	0.082	0.47	0.104	0.37	−0.219	0.17	−0.208	0.18	−0.145	0.19	−0.066	0.56	0.01	0.93	0.027	0.85
Q2.3	0.233	0.04	0.047	0.68	−0.003	0.97	−0.159	0.16	0.002	0.99	0.068	0.56	-.346	0.03	−0.184	0.23	0.062	0.58	0.116	0.310	0.007	0.95	−0.037	0.79
Q2.4	0.108	0.34	−0.005	0.97	−0.128	0.26	−0.044	0.67	0.115	0.31	0.077	0.51	−0.175	0.28	−0.107	0.48	−0.057	0.61	0.013	0.91	0.013	0.910	−0.059	0.68
Q2.5	0.396	0.00	0.022	0.85	0.047	0.67	−0.191	0.08	−0.151	0.18	−0.149	0.19	−0.204	0.21	−0.105	0.49	0.155	0.17	0.131	0.25	−0.223	0.04	0.143	0.32
**Self-confidence**																								
Q2.6	0.336	0.00	0.005	0.97	0.016	0.87	−0.2	0.07	−0.11	0.33	−0.214	0.06	0.005	0.98	0.038	0.81	0.251	0.02	0.313	0.00	−0.264	0.020	0.286	0.04
Q2.7	0.184	0.10	−0.067	0.55	−0.186	0.09	−0.15	0.18	−0.008	0.94	−0.046	0.69	−0.172	0.29	−0.19	0.22	0.068	0.55	0.097	0.39	−0.115	0.31	0.034	0.81
Q2.8	0.320	0.00	−0.191	0.09	-.269	0.01	−0.177	0.11	0.029	0.79	−0.161	0.16	−0.068	0.68	−0.141	0.36	−0.035	0.76	0.063	0.58	−0.227	0.04	0.269	0.06
Q2.9	0.328	0.00	−0.007	0.95	0.027	0.81	−0.164	0.14	−0.01	0.93	−0.046	0.68	−0.139	0.39	−0.072	0.64	0.094	0.400	0.149	0.190	−0.153	0.18	0.102	0.48
Q2.10	0.168	0.14	−0.106	0.34	−0.001	0.99	0.055	0.63	0.072	0.52	0.105	0.36	−0.216	0.18	−0.176	0.250	−0.128	0.26	−0.143	0.21	−0.075	0.51	0.031	0.83
Q2.11	0.223	0.05	0.231	0.04	−0.075	0.50	−0.104	0.36	0.022	0.84	−0.017	0.88	−0.036	0.83	−0.046	0.77	−0.041	0.71	0.019	0.87	0.029	0.79	0.198	0.16
Q2.12	0.333	0.00	0.072	0.52	0.02	0.86	−0.165	0.14	−0.09	0.43	−0.17	0.14	0.027	0.87	0.064	0.68	0.063	0.58	0.162	0.15	−0.340	0.00	0.394	0.00
Q2.13	−0.211	0.06	−0.225	0.04	−0.13	0.25	0.187	0.09	0.203	0.07	−0.012	0.92	−0.232	0.160	−0.316	0.04	−0.152	0.18	−0.137	0.23	0.031	0.79	−0.119	0.041

EMC, emergency medical service.

Note: *r* is the Spearman’s rank correlation coefficients.

*p* < 0.1; *p* < 0.05; *p* < 0.01 are significant at 10%, 5% and 1%, respectively.

#### Demographic characteristics and self-confidence variables

Table 3 shows the associations between the paramedic students’ demographic characteristics and the self-confidence variables. Age was noted to be moderately, positively and significantly associated with the variable, ‘I am confident that I am mastering the content of the simulation activity that my lecturers presented to me’ (*r* = 0.336, *p* = 0.00). The variable, ‘I am confident that I am acquiring the skills and knowledge required from this simulation to perform necessary tasks in a clinical setting’, was also noted to be significantly, moderately and positively associated with age (*r* = 0.320, *p* = 0.00). Similar moderate, positive and statistically significant associations between age and the following confidence variables were reported: ‘My lecturers used helpful resources to teach the simulation’ (*r* = 0.328, *p* = 0.00) and ‘I know how to use simulation activities to learn critical aspects of clinical skills’ (*r* = 0. 333, *p* = 0.00).

With regard to gender (male), a positive, weak and statistically significant association was noted with the variable, ‘I know how to get help when I do not understand the concepts covered in the simulation’ (*r* = 0. 231, *p* = 0.04) (Table 3). Race (Africans) showed a negative, weak and significant association with the self-confidence variable, ‘I am confident that I am developing the skills and obtaining the required knowledge from this simulation to perform necessary tasks in a clinical setting’ (*r* = –0.269, *p* = 0.02). Similarly, a significant association was found between ‘EMS-related training undertaken’ and the self-confidence variable, ‘I am confident that I am mastering the content of the simulation activity that my lecturers presented to me’ (*r* = 0.313, *p* = 0.00). The association between the ‘EMS-related training not offered by the EMC programme’ and the self-confidence variable, ‘I am confident that I am mastering the content of the simulation activity that my lecturers presented to me’ was reported to be positive and significant (*r* = 0.251, *p* = 0.02). The self-confidence variable, ‘I am confident that I am mastering the content of the simulation activity that my lecturers presented to me’ showed a statistically significant association with the work and study variable (*r* = –0. 264, *p* = 0.02).

The work and study variable also showed statistically significant associations with the following self-confidence variables, ‘I am confident that I am developing the skills and obtaining the required knowledge from this simulation to perform necessary tasks in a clinical setting’ (*r* = –0. 227, *p* = 0.04) and ‘I know how to use simulation activities to learn critical aspects of clinical skills’ (*r* = –0. 340, *p* = 0.00). Positive and significant associations were found between the work description variable and the self-confidence variables, ‘I am confident that I am mastering the content of the simulation activity that my lecturers presented to me’ (*r* = 0.286, *p* = 0.04), and ‘I know how to use simulation activities to learn critical aspects of clinical skills’ (*r* = 0. 394, *p* = 0.00).

#### Predictors of the paramedic students’ satisfaction with and self-confidence in clinical simulation

Table 4 shows the paramedic students’ demographic characteristics that may predict their satisfaction with self-confidence in the clinical simulation activities at the Durban University of Technology. An ordinal logistic regression analysis was performed and a significant improvement in the fit of the model was revealed for the self-confidence variable, ‘It is the lecturer’s responsibility to tell me what I need to learn of the simulation activity content during class time’ (Question 2.13) and the paramedic students’ demographic characteristic (*Χ*^2^ (12) = 21.747, *p* = 0.40). Amongst the independent variables, only ‘EMS related training undertaken’ was significantly associated with the self-confidence variable item, ‘It is the lecturer’s responsibility to tell me what I need to learn of the simulation activity content during class time’ (Question 2.13) (Odds ratio [OR] = 1.116, *p* = 0.002). Apart from gender, age and race, the rest of the independent variables had OR values greater than one; ‘marital status’ (OR = 1.395, confidence interval [CI] = 920–2.117), ‘admission based on Recognition of prior learning (RPL)’ (OR = 1.155, CI = 0.854–1.562), ‘simulation experience’ (OR = 1.342, CI = 0.831–2.167), ‘years of simulation experience’ (OR = 1.048, CI = 0.819–1.342), ‘EMS training before joining the EMC Programme’ (OR = 1.022, CI = 0.913–1.145), ‘EMS related training not offered by the EMC programme’ (OR = 1.067, CI = 0.644–1.768), ‘EMS related training undertaken’ (OR = 1.116, CI = 1.041–1.197), ‘work and study’ (OR = 1.238, CI = 0.745–2.054), and ‘work description’ (OR = 1.300, CI = 0.963–1.779).

**TABLE 4:** Factors associated with self-confidence variables.

Characteristics	Ordinal logistic analysis		*p*
	Odds ratio (OR)	95% CI	
Age	0.973	0.837–1.131	0.723
Gender	0.966	0.744–1.254	0.795
Race	0.972	0.872–1.084	0.615
Marital status	1.395	0.920–2.117	0.117
Admission based on RPL	1.155	0.854–1.562	0.349
Simulation experience	1.342	0.831–2.167	0.229
Years of simulation experience	1.048	0.819–1.342	0.709
EMS training before joining the EMC programme	1.022	0.913–1.145	0.705
EMS related training not offered by the EMC programme	1.067	0.644–1.768	0.801
EMS related training undertaken	1.116	1.041–1.197	**0.002**
Work and study	1.238	0.745–2.054	0.410
Work description	1.300	0.963–1.779	0.085

Note: Bold value indicate 1% significance level of confidence.

EMC, emergency medical service; CI, confidence intervals.

## Discussion

Paramedics are required by their professional bodies to acquire specific levels of competencies for the provision of safe and effective care to patients. Thus, the development of clinical skills of paramedic students before embarking on the care of real patients is of great importance. Innovative technologies are used in healthcare education for ensuring this. Clinical simulation has emerged as a significant instructional method for creating conducive learning environments, improving satisfaction and confidence in learning, and enhancing knowledge and skill acquisition (Rodriguez et al. 2017). This is a function of the view that clinical simulation allows students to repeatedly practise skills and procedures, and lecturers to offer prompt and specific feedback for every mistake the students make until they master those specific skills and procedures (Omer 2016). Acknowledging this, clinical simulation exposes students to clinical events without putting the health of real patients at risk (McBride & Waldrop 2018).

This study investigated the paramedic students’ self-confidence and satisfaction with the clinical simulation of an EMC programme at the Durban University of Technology in South Africa. Its outcome revealed that most of the paramedic students were satisfied with the clinical simulation of this institution, as about 63% of them reported that the teaching methods used were helpful for their learning. Added to this, approximately 66% of the paramedic students claimed that the simulation was well resourced with activities for learning, and about 66% of them noted that the teaching materials used in the simulation assisted them to learn clinical skills. Whilst a small proportion of the paramedic students were either uncertain or dissatisfied with the clinical simulation, the high proportion of satisfaction reported here indicates that this mode of teaching at the Durban University of Technology was adequate for the learning of clinical skills. This is consistent with the outcome of a systematic review by Sendir and Degan (2015). It notes that students are generally satisfied with clinical simulation, as it promotes knowledge and skill acquisition.

The emphasis on simulation education is often on knowledge and skill development, and enhancement of critical thinking and clinical judgement, including their application (Karkada et al. 2019). The achievement of these attributes can be influenced by students’ self-confidence. In McCabe, Gilmartin and Goldsamt’s (2016) view, self-confidence is a person’s belief in his or her ability to carry out a specific task effectively in a particular situation. Taking this into consideration, self-confidence is a skill that can be learnt and practised. Thus, assessing students’ self-confidence in clinical simulation is critical for ensuring patients’ safety, promoting students’ learning and satisfaction with the decisions they make. The results of this study point out that clinical simulation could enhance the paramedic students’ self-confidence to perform clinical skills, a view echoed by Williams et al (2016). Over half of the paramedic students (approximately 57%) claimed that they were confident that the clinical simulation could enable them to acquire the skills and knowledge required to perform tasks in clinical settings. About 68% of them were confident that they knew how to use clinical simulation to learn clinical skills. The high proportions of self-confidence reported suggests that clinical simulation can serve to reinforce self-confidence (Laure et al. 2015) and self-confidence in turn, can serve as an important determinant of quality care provision. However, a small proportion of the paramedic students reported a lack of self-confidence in the clinical simulation activities. The differences in the proportions of self-confidence reported could be attributed to the variations in the paramedic students’ demographic characteristics such as their experiences of clinical simulation and the feelings of anxiety and stress that may be evoked by the scenarios (Weller 2004).

This study reported associations between the paramedic students’ demographic characteristics and the satisfaction and self-confidence variables. A moderate positive and significant association was obtained between age and the satisfaction variable, ‘The way my lecturer taught the simulation was suitable to the way I learn’. Though weak, age was positively and significantly associated with the satisfaction variable, ‘I enjoyed how my lecturer delivered the simulation training’. These outcomes suggest that as the paramedic students age, their satisfaction with clinical simulation tends to increase. Moderate positive and significant associations were also reported between age and the self-confident variables, ‘I am confident that I am mastering the content of the simulation activity that my lecturers presented to me’, ‘I am confident that I am developing the skills and obtaining the required knowledge from this simulation to perform necessary tasks in a clinical setting’ and ‘I know how to use simulation activities to learn critical aspects of clinical skills’. The building of confidence in simulation learning evidenced in the results is crucial, considering its role in facilitating learning and strengthening students’ ability to utilise skills and knowledge safely in clinical practice, a view echoed by Sarman and Pardi (2019).

Moderate negative and significant associations were found between years of simulation experience and the following satisfaction variables, ‘The teaching methods used in this simulation were helpful and effective’, and ‘I enjoyed how my lecturer delivered the simulation training’. This suggests that as the paramedic students gain experience of clinical simulation, their satisfaction with this mode of facilitation of learning decreases. Experiences of feelings of dissatisfaction amongst students of any profession are undesirable, but they are even more undesirable if experienced by students of healthcare professions like paramedics (Sarman & Pardi 2019). The primary aim of paramedic students in clinical simulation is to gain the skills and knowledge required for the safe provision of quality care to patients (Rodriguez et al. 2017). This goal is unlikely to be realised if dissatisfaction with simulation exists amongst this student population.

The work and study variable was found to have a negative weak but significant association with the satisfaction variable, ‘The way my lecturer(s) taught the simulation was suitable to the way I learn’. Although negative, it also showed weak and moderate significant relationships with the following self-confidence variables, ‘I am confident that I am mastering the content of the simulation activity that my lecturers presented to me’ and ‘I know how to use simulation activities to learn critical aspects of clinical skills’, respectively. These outcomes seem to point out that the paramedic students in employment at a clinical setting (e.g. ambulance services) are more likely, over time, to be less confident and satisfied with clinical simulation (Williams et al. 2016). This could be attributed to the view that the clinical skills, including critical thinking and decision-making they learn at work could be similar to those taught in clinical simulation. Therefore, clinical simulation might not be perceived as a forum for the advancement of clinical skills and knowledge.

The ordinal logistic regression analysis result revealed a significant association between the paramedic students’ demographic characteristic ‘emergency medical service related training undertaken’ and the self-confidence variable, ‘It is the lecturer’s responsibility to tell me what I need to learn of the simulation activity content during class time’. This relationship indicates that the paramedic students who have had emergency medical service training were less likely to assume responsibility for their learning. Healthcare education in South Africa and Africa in general mainly relies on didactic approaches to teaching and learning (e.g. lectures) where lecturers are the providers of knowledge and instructions and students the recipient of the same (Couper et al. 2018). It is therefore not surprising for the paramedic students to rely on the lecturers for instructions on what they were required to learn. Such reliance generates boredom and limits students’ participation and ability to ask questions to fill gaps in their understanding of concepts taught. Teaching methods, such as role-play and group work that go beyond information giving and engage the whole person have long been associated with attitude change and enhancement of students’ participation in their learning (Knowles 1990). The use of such teaching methods should therefore precede clinical simulation sessions given their role in enabling students to assume responsibility for their learning.

## Limitations

This is a quantitative study that is not free from limitations. It utilised a convenience sampling method for the selection of its participants, which led to a sample size of 83 paramedic students. The study was conducted in one university. Paramedic students of other universities of technology may have different experiences of clinical simulation. Given this and the small sample size, the results of this may not be generalised to other settings.

## Implications for practice and research

Simulation is a practice andragogy that aims at bridging the theory-practice gap, enhancing learners’ clinical skills, confidence and competency. The outcome of this study revealed that the paramedic students were generally confident and satisfied with clinical simulation. However, it would be erroneous to assume that the high proportions of confidence and satisfaction revealed in this study could translate into increases in clinical competency. Thus, lecturers need to devise strategies for assessing learners’ competencies and ensuring that they attain acceptable levels of competencies in clinical skills during simulation sessions. Some of the paramedic students expressed dissatisfaction with clinical simulation and this is a concern, as feelings of dissatisfaction may interfere with concentration that in turn may impede learning. Hence, the learning needs and styles of the paramedic students need to be taken into account by lecturers when planning for simulation sessions. Added to this, lecturers may consider working in partnership with third-year paramedic students as co-facilitators since they were reported to be less satisfied with clinical simulation. Such a stance would enable them to feel valued, respected and increase their satisfaction levels with this mode of facilitation.

Given that this study was conducted only in one setting, future studies need to be conducted in multiple settings and to include qualitative methods. The rationale here is to explore the experiences of learners in relation to clinical simulation. Future studies need to investigate the associations between the self-confidence and the satisfaction variables, and the effects of the demographic characteristics on the self-confidence and the satisfaction variables. Doing so would generate more insight into this phenomenon and assist lecturers in designing strategies for enhancing students’ satisfaction and confidence in clinical simulation. It would also be critical for future studies to assess the relationship between simulation methods and competence in practice.

Doing so would enable researchers to establish whether simulation as an instructional tool would ensure the transfer of learnt skills to clinical practice.

## Conclusion

Clinical simulation is an effective teaching method for improving the competencies of paramedic students. It increases the paramedic students’ satisfaction and self-confidence in the application of clinical skills without risking patient safety and health. It is envisaged that the use of third-year paramedic students as co-facilitators would contribute to increase their satisfaction and self-confidence in clinical simulation. Increasing the paramedic students’ self-confidence and satisfaction can improve the quality of care they provide to patients. Thus, the findings of this study are of practical utility for both students and lecturers.
